# Effects of Ulinastatin on Inflammation Response and Lung Tissue Injury in Deep Hypothermic Circulatory Arrest

**DOI:** 10.1093/icvts/ivaf177

**Published:** 2025-07-29

**Authors:** Qiang Hu, Yuan Teng, Yuan Yuan, Guodong Gao, Bingyang Ji

**Affiliations:** Department of Cardiopulmonary Bypass, National Center for Cardiovascular Diseases & Fuwai Hospital, Peking Union Medical College & Chinese Academy of Medical Sciences, 10037 Beijing, China; Department of Cardiopulmonary Bypass, National Center for Cardiovascular Diseases & Fuwai Hospital, Peking Union Medical College & Chinese Academy of Medical Sciences, 10037 Beijing, China; Department of Cardiopulmonary Bypass, National Center for Cardiovascular Diseases & Fuwai Hospital, Peking Union Medical College & Chinese Academy of Medical Sciences, 10037 Beijing, China; Department of Cardiopulmonary Bypass, National Center for Cardiovascular Diseases & Fuwai Hospital, Peking Union Medical College & Chinese Academy of Medical Sciences, 10037 Beijing, China; Department of Cardiopulmonary Bypass, National Center for Cardiovascular Diseases & Fuwai Hospital, Peking Union Medical College & Chinese Academy of Medical Sciences, 10037 Beijing, China

**Keywords:** cardiopulmonary bypass, acute respiratory insufficiency, deep hypothermic circulatory arrest, systemic inflammatory response, ischaemia-reperfusion, ulinastatin

## Abstract

**Objectives:**

Deep hypothermic circulatory arrest (DHCA) is known to trigger a systemic inflammatory response and ischaemia-reperfusion injury, leading to exacerbated lung dysfunction. Ulinastatin (UTI) is a commonly used anti-inflammatory drug in clinical settings, but its protective effects may vary depending on the timing and dosage.

**Methods:**

A rat model of DHCA was established, and 2 different doses of UTI (5/10 × 10^4^ U/kg; low/high dose) were administered. We measured the levels of inflammatory factors using enzyme-linked immunosorbent assay kits and assessed the functional indicators of lung tissue injury. All rats (*n* = 18) underwent the standard cardiopulmonary bypass (CPB) procedure with DHCA.

**Results:**

Following rewarming, the levels of interleukin-6 (IL-6), IL-10, tumour necrosis factor (TNF)-α, and neutrophil elastase 2 (ELA-2) gradually increased in rats exposed to DHCA. Compared to the DHCA group, both the UTI groups exhibited significant reductions in IL-6 (DHCA vs DHCA+UTI-H, 8931.68 ± 650.31 vs 2498.05 ± 552.16), TNF-α (DHCA vs DHCA+UTI-H, 633.74 ± 74.53 vs 221.19 ± 31.63), and ELA-2 (DHCA vs DHCA+UTI-H, 4.94 ± 0.49 vs 3.29 ± 0.34), while remarkably increased the IL-10 (DHCA vs DHCA+UTI-H, 975.04 ± 110.33 vs 3081.27 ± 554.10) levels 4 hours after weaning from CPB (all *P *< 0.05). Interestingly, the high dose of UTI demonstrated a dose-dependent inhibition of inflammation. Meanwhile, we found that UTI contributed to maintain haemodynamic stability, improve tissue perfusion, and reduce hypoxia, as evidenced by elevated heart rate, blood pressure, haematocrit and oxygenation index, and decreased glucose and lactate. Reduced pathological changes in lung histopathology were also observed after UTI intervention, especially in 10 × 10^4^ U/kg group.

**Conclusions:**

This study revealed that administration of low to high doses of UTI during DHCA could reduce the release of inflammatory factors, exert anti-inflammatory effects, and alleviate lung injury.

## Graphical Abstract

**Figure:**
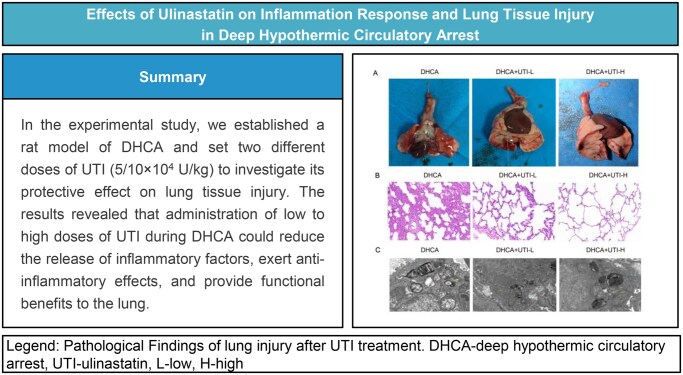

## INTRODUCTION

Acute respiratory insufficiency (ARI) following cardiopulmonary bypass (CPB) is a common condition known as post-perfusion lung syndrome.^1^ At present, there is no specific treatment for ARI, and the mortality rate is as high as 45% in the general population, which escalates to 80% in cardiac surgery patients.^2–5^ This condition is particularly severe in patients undergoing total arch replacement surgery with deep hypothermic circulatory arrest (DHCA). Studies have shown that the inflammatory response caused by ischaemia-reperfusion (IR) injury during DHCA can increase alveolar capillary permeability, leading to the development of ARI.^6–8^ This not only prolongs the weaning time and intensive care unit (ICU) stay time^9^ but also increases the risk of nosocomial infection.^10^ Therefore, identifying suitable compounds to attenuate inflammation and improve prognosis following DHCA is urgently needed.

Ulinastatin (UTI) is a protease inhibitor derived from healthy human urine. It has been used clinically to inhibit various serine proteases and stabilize lysosomal membranes and cell membranes.^11^ UTI is mainly metabolized through the kidney. In healthy males, the blood concentration of UTI decreases linearly within 3 hours after intravenous injection, with an elimination half-life (t_1/2_) of 40 min. Approximately 24% of UTI is excreted in the urine within 6 hours after intravenous administration.^12^ UTI is effective in treating inflammatory diseases conditions, including acute pancreatitis and sepsis.^13–15^ In heart surgery, it has been demonstrated to decrease postoperative bleeding, preserve platelet function, prevent excessive fibrinolysis, and lessen inflammation.^16^ Additionally, available data have shown that UTI attenuates acute pulmonary injury by affecting the activities of nuclear factor-kB and its relevant inflammatory mediators.^17^^,^^18^ However, the short half-life of UTI and its dose-dependent suppression of systemic inflammatory response (SIRS) following pulmonary IR injury necessitate careful consideration of dosage and timing in its application. Moreover, the effects of high doses of UTI on changes in lung protection remains poorly understood, therefore, whether higher doses of UTI can bring more benefits still needs to be further explored.

In this research, we hypothesized that high-dose UTI would exert a stronger anti-inflammatory effect and alleviate lung injury compared to low-dose UTI in a rat model of DHCA. To test this hypothesis, we administered low and high doses of UTI to investigate its safeguarding impact on the lung tissue injury and to determine the most effective dosage.

## METHODS

### Animals and groups

The animal experiment received approval from the Animal Experimental Ethics Committee of Fuwai Hospital (FW-2022-0001) and adhered to the regulations of International Laboratory Animal Management Regulations. The study was reported in accordance with ARRIVE guidelines. At the Fuwai Animal Center, male Sprague Dawley rats, aged 12 to 14 weeks and weighing 450 to 550 g, were kept under standard laboratory conditions. They were obtained from HFK Bioscience, China. The rats were kept in stables with a temperature of 22°C, a relative humidity of 55%, and a 12/12 hour day/night cycle. They were provided with free access to both food and water.

Three groups were formed by randomly dividing a total of 18 rats: the DHCA group (*n* = 6), the 5 × 10^4^ U/kg UTI-treated DHCA group (DHCA+UTI-L, *n* = 6), and the 10 × 10^4^ U/kg UTI-treated DHCA group (DHCA+UTI-H, *n* = 6). The powder for each group was dissolved in 2 mL of normal saline. The dose was divided equally, with half added to the CPB priming solution and the other half administered post-rewarming. This exploratory study used empirically determined sample sizes common in preclinical DHCA research.^19^^,^^20^

### CPB and DHCA procedure

The CPB and DHCA procedures followed the methods reported previously.^21^ Briefly, the rats underwent endotracheal intubation under 2%-3% sevoflurane anaesthesia. After tracheal intubation with a 16 G arterial cannula, mechanical ventilation was initiated using a ventilator (with an oxygen concentration of 60%, tidal volume of 8 mL/kg, inspiratory-to-expiratory ratio of 1:1, and a frequency of 60 breaths per minute). Continuous monitoring of mean arterial blood pressure (MAP) was achieved by cannulating the left femoral artery, while the right external jugular vein and tail artery were catheterized in order to establish CPB. After intravenous administration of 500 IU/kg heparin via the right external jugular vein, CPB was started using a system that comprised a modified reservoir (based on Murphy’s dropper), a roller pump, a heat exchanger (Xijian, Xian, China), a membrane oxygenator (Xijian, Xian, China), connecting tubes, and a water tank.^22^

**Figure 1.**
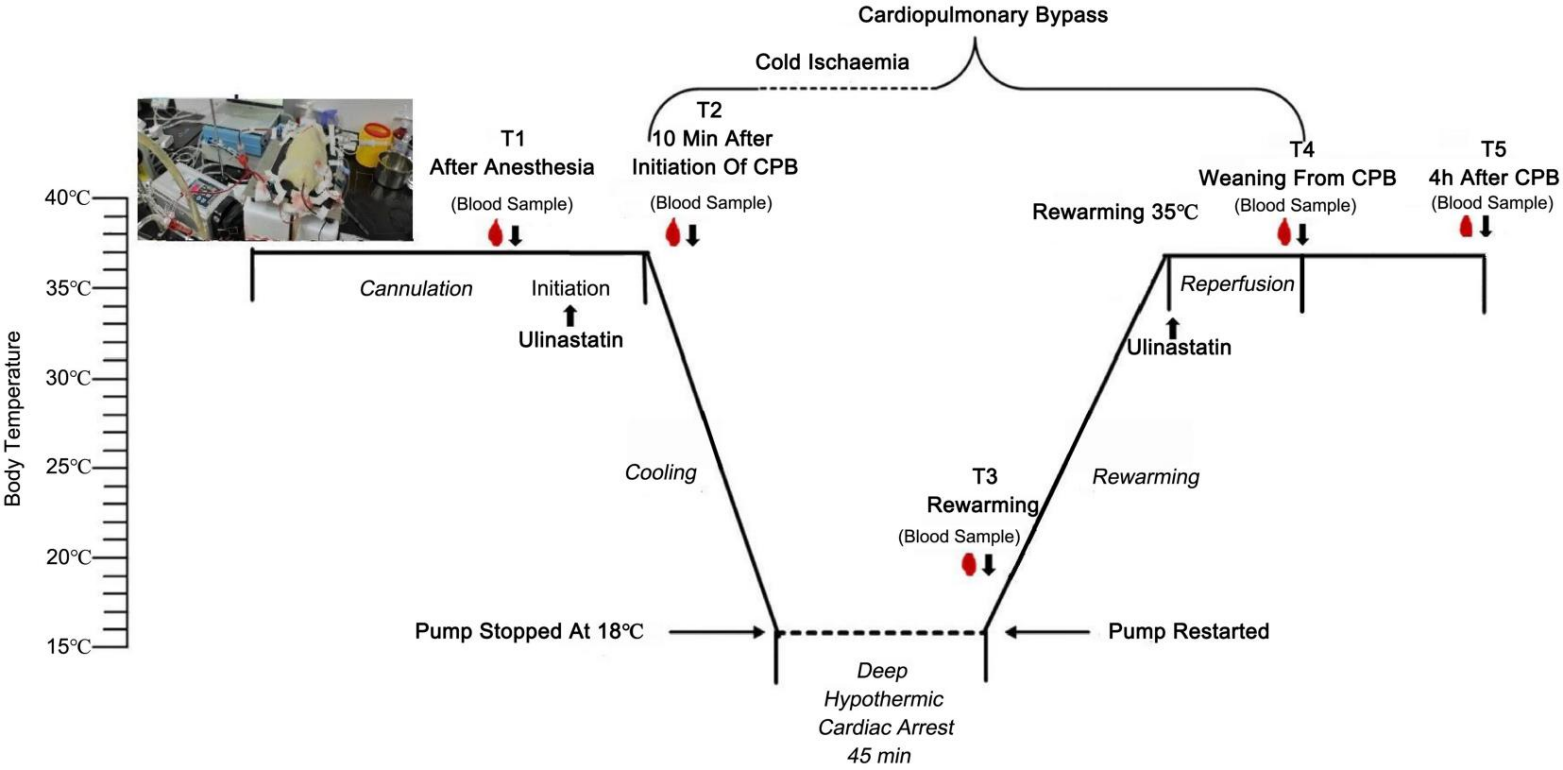
Schematic Illustration of the Experimental Time and Temperature Flow. T1-T5 represent predefined time points for blood sampling. Abbreviations: T1: after anesthesia; T2: 10 min after initiation of CPB; T3: rewarming; T: weaning from CPB; T5: 4 h after CPB; CPB: cardiopulmonary bypass

In the CPB circuit, the priming solution was made of 12 mL of 6% hydroxyethyl starch plus 2 mL of normal saline with 150 IU of heparin. CPB was initiated at a flow rate of 160-180 mL/kg/min for the first 10 min, followed by systemic cooling to achieve a target deep temperature of 18°C. During the cooling period, the acid-base balance was maintained using alpha-stat strategy. The induction of DHCA was achieved by draining blood into the reservoir, lasting around 45 min, as indicated by a MAP of 0. The rewarming phase involved gradually increasing the rectal temperature to 35°C while recovering circulation. During the entire CPB process, when the volume was insufficient, Gelofusine was supplemented at 1-2 mL each time. After rewarming to 30°C, norepinephrine and dopamine (**Table S1**) were used to maintain the mean arterial pressure ≥ 60 mmHg. Once the rat’s vital signs stabilize, the roller pump speed was gradually reduced until the machine was stopped. The CPB overall time was 212-224 min. Following a 4-hour period of anaesthesia with mechanical ventilation support, the rats were humanely euthanized through cervical dislocation. Formaldehyde was used to promptly preserve the lung specimens after collection. A timeline illustrating the experimental procedures, including drug administration and sample collection, was presented in **Figure 1**. During the operation, continuous monitoring was conducted for baseline physiological parameters, including MAP, heart rate, and rectal temperature.

### Blood gas analysis

Blood gas analysis (i-STAT, Chicago, IL, USA) involved collecting 0.2 mL arterial blood samples at 5 distinct times: postanaesthesia (T1), 10 min after starting CPB (T2), during the rewarming phase (T3), after CPB weaning (T4), and 4 hours post-CPB (T5).^22^ Adjustments to ventilator parameters and electrolyte levels were made according to the rats’ blood gas results.

### Enzyme-linked immunosorbent assay

Serum was quantified from blood samples collected at T2, T4, and T5, with interleukin 6 (IL-6), IL-10, tumour necrosis factor-α (TNF-α), and neutrophil elastase 2 (ELA-2) measured using enzyme-linked immunosorbent assay kits.

### Haematoxylin and eosin staining and transmission electron microscopy

Following the euthanasia of the rats, lung sections with dimensions of 1 mm thick by 2 mm by 2 mm were swiftly collected. These samples were fixed in 4% formaldehyde for 2 hours and then stored in a refrigerator at temperatures ranging from 0 to 4°C. After dehydration, embedding, slicing, and haematoxylin and eosin (HE) staining, tissue morphology was examined microscopically.

Ultrastructure was observed via transmission electron microscopy (TEM). The samples (1 mm^3^) were fixed in 2.5% phosphate-buffered glutaraldehyde for 20 min at 37°C and post-fixed in 1% osmium tetroxide (Sigma-Aldrich Co. LLC., USA) in water at 37°C for 30 min followed by gradient dehydration for each time 15 min with ethanol and acetone (50% ethanol, 70% ethanol, 90% ethanol, 90% acetone, 100% acetone) at 37°C. After embedding and sectioning (60 nm), the samples were double stained with uranium-lead. Images were acquired by TEM and analysed using Image-Pro Plus 6.0 software.

### Statistical analysis

The experimental data were statistically analysed with GraphPad Prism 9.0 (GraphPad San Diego, CA, USA) and SPSS 27.0 (SPSS Inc, Chicago, IL). The normality of continuous variables was assessed using the Shapiro-Wilk test. The results were expressed as mean ± standard deviation or median and quartile 1 to quartile 3 (Q1-Q3), as appropriate (**Tables S2-S4**). One-way analysis of variance (ANOVA) followed by Bonferroni’s post hoc test or Kruskal-Wallis test followed by Dunn’s post-hoc test was used to compare differences in haemodynamic parameters, inflammatory factors, and laboratory variables of organ function at each time point. Using R Studio (version 4.5.0) with the lme4 package, perform an analysis of the transformed (log-transformed) data using a linear mixed-effects model (LMEM). A *P*-value < 0.05 (2-tailed) indicated statistical significance.

## RESULTS

### Haemodynamic changes

**Figure 2.**
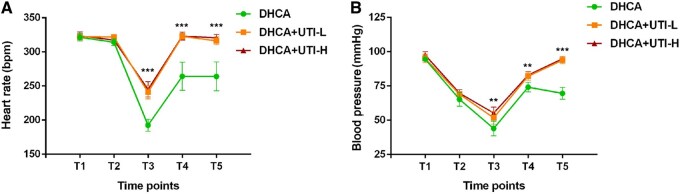
Changes of Haemodynamics in DHCA, DHCA+UTI-L, and DHCA+UTI-H Groups of Rats. HR (A) and BP (B) were monitored throughout the experiment. **P < 0.01, ***P < 0.001 indicated DHCA group vs UTI groups. All *P*-values represent between-group comparisons at the specified time points. Abbreviations: DHCA: deep hypothermic circulatory arrest; UTI: ulinastatin; UTI-L: low dose UTI-treated; UTI-H: high dose UTI treated

The basic haemodynamic parameters were reported in **Figure 2**. The initial 10 min after establishing CPB served as an adaptation stage for rats to the CPB circuit. During this process, the heart rate and blood pressure were similar among the 3 groups. As the rats cooled down, their heart rate and blood pressure gradually decreased but subsequently recovered upon rewarming. The heart rate of the DHCA group was slower than that of the other 2 groups at T3, T4, and T5 (*P *< 0.001). The blood pressure of the DHCA+UTI-L and DHCA+UTI-H was higher than that of the DHCA group at T3 and T4 (*P *< 0.01) and T5 (*P *< 0.001). The LMEM analysis revealed statistically significant differences in heart rate between the groups treated with DHCA+UTI-L and DHCA+UTI-H compared with the DHCA group (T3: Estimate: 0.22 95% CI [0.16, 0.28] *P *< 0.001, 0.24 [0.18, 0.29] *P *< 0.001; T4: 0.20 [0.14, 0.26] *P *< 0.001, 0.20 [0.14, 0.26] *P *< 0.001; T5: 0.18 [0.12, 0.24] *P *< 0.001, 0.19 [0.13, 0.25] *P *< 0.001, respectively). Similarly, there are similar results in terms of blood pressure (**Table S5**).

### Blood count and metabolites

**Figure 3.**
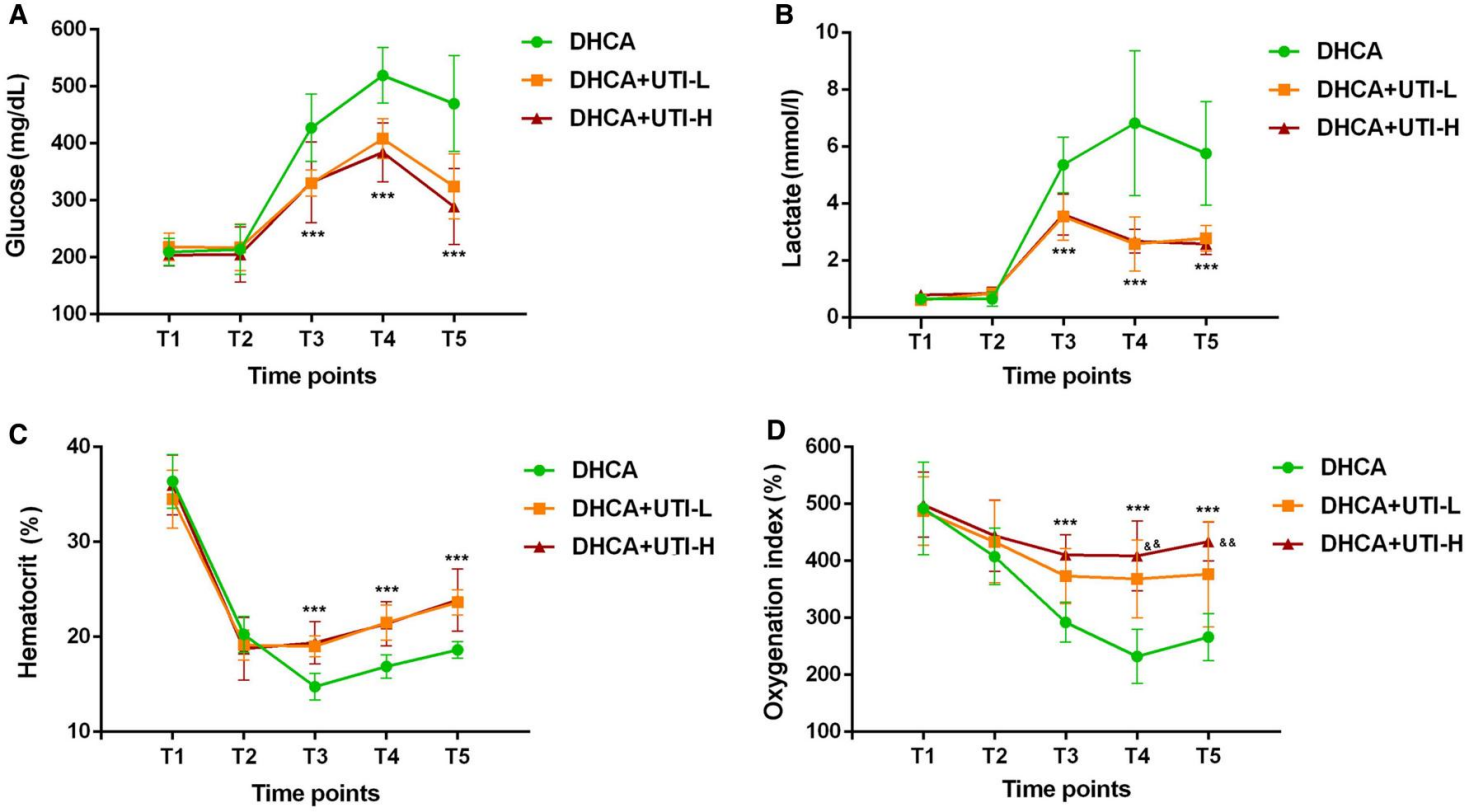
Blood Gas Analysis in DHCA, DHCA+UTI-L, and DHCA+UTI-H Groups of Rats. Glu (A), Lac (B), Hct (C), and OI (D) were measured at predefined time-points for 3 groups of rats. ***P < 0.001 indicated DHCA group vs UTI groups. ^&&^*P *< 0.01 indicated DHCA+UTI-L group vs DHCA+UTI-H group. All *P*-values represent between-group comparisons at the specified time points. Abbreviations: DHCA: deep hypothermic circulatory arrest; UTI-L: low dose ulinastatin-treated; UTI-H: high dose ulinastatin-treated

The results of blood gas analyses were shown in **Figure 3**. During the first 10 min of CPB establishment, the rats underwent an adaptation process, and there were no significant differences in blood gas results among the 3 groups at this time point. During DHCA, ischaemia and hypoxia led to the accumulation of anaerobic metabolites. After rewarming, the concentrations of glucose and lactate significantly increased (**Figure 3A** and **B**). With sufficient blood flow support, the concentration of glucose and lactate showed a decreasing trend. The concentrations of glucose and lactate in the DHCA group were significantly higher than those in the other 2 groups at T3, T4, and T5 (*P *< 0.001). The LMEM analysis showed that compared to the DHCA group, both the DHCA+UTI-L and DHCA+UTI-H groups had statistically significant differences in terms of glucose and lactate (for glucose, T5: −0.41 [−0.67, −0.15] *P *= 0.003, −0.48 [−0.74, −0.21] *P *< 0.001, respectively. for lactate, T4: −0.96 [−1.40, −0.52] *P *< 0.001, −1.10 [−1.54, −0.66] *P *< 0.001; T5: −0.65 [−1.09, −0.21] *P *= 0.005, −0.98 [−1.42, −0.54] *P *< 0.001, respectively).

At the beginning of CPB, the haematocrit and oxygenation index of the 3 groups significantly decreased and gradually increased after rewarming (**Figure 3C** and **D**). The haematocrit and oxygenation index in the DHCA group were significantly lower than those in the other 2 groups at T3, T4, and T5 (*P *< 0.001). The oxygenation index of the DHCA+UTI-L group was significantly lower than that of the DHCA+UTI-H group at T4 and T5 (*P *< 0.01). Both low and high doses differed significantly from the control group in haematocrit and oxygenation index based on LMEM analysis (for haematocrit, T3: 0.31 [0.18, 0.44] *P *< 0.001, 0.28 [0.15, 0.42] *P *< 0.001; T4: 0.29 [0.16, 0.43] *P *< 0.001, 0.24 [0.11, 0.38] *P *< 0.001; T5: 0.29 [0.16, 0.43] *P *< 0.001, 0.25 [0.12, 0.38] *P *< 0.001, respectively. for oxygenation index, T3: 0.23 [0.01, 0.44] *P *= 0.042, 0.31 [0.09, 0.52] *P *= 0.007; T4: 0.47 [0.26, 0.69] *P *< 0.001, 0.56 [0.34, 0.77] *P *< 0.001; T5: 0.33 [0.12, 0.55] *P *= 0.003, 0.48 [0.26, 0.69] *P *< 0.001, respectively).

### Inflammatory factor changes

**Figure 4.**
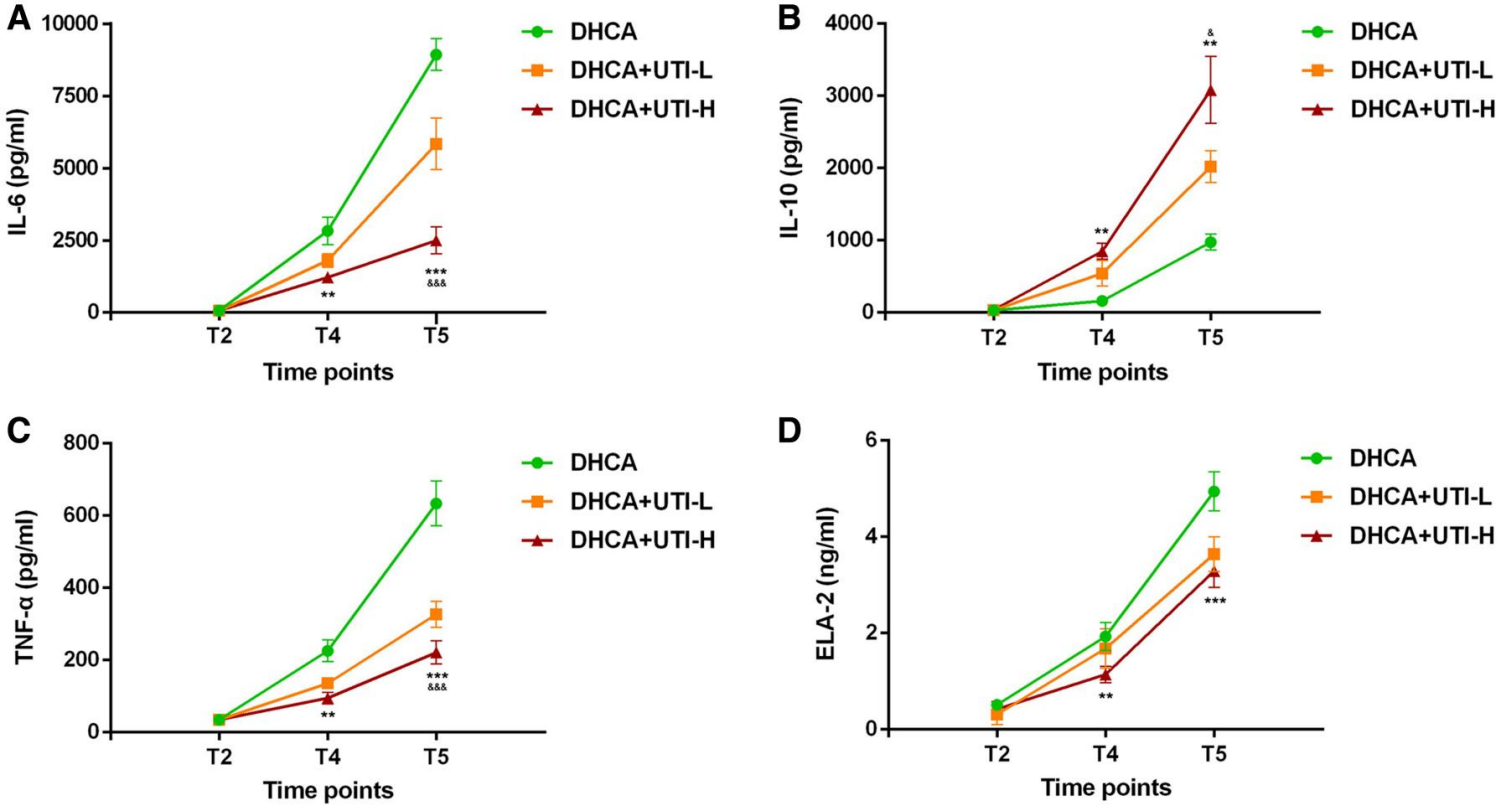
Changes in Inflammatory Factors in DHCA, DHCA+UTI-L, and DHCA+UTI-H Groups of Rats at Different Time. (A) IL-6, (B) IL-10, (C) TNF-α, and (D) ELA-2 were measured by ELISA. **P < 0.01, ***P < 0.001 indicated DHCA group vs UTI groups; ^&^*P *< 0.05, ^&&&^*P *< 0.001 indicated DHCA+UTI-L group vs DHCA+UTI-H group. All *P*-values represent between-group comparisons at the specified time points. Abbreviations: DHCA: deep hypothermic circulatory arrest; UTI: ulinastatin; UTI-L: low dose UTI treated; UTI-H: high dose UTI-treated; IL-6: interleukin 6; IL-10: interleukin 10; TNF-α: tumour necrosis factor-α; ELA-2: elastase 2

Following the DHCA procedure, IL-6, IL-10, TNF-α, and ELA-2 concentrations in DHCA rats increased gradually (**Figure 4** and **Table S6**). Different doses of UTI had varying effects on the inhibition of inflammatory factors. At T5, the concentrations of IL-6, TNF-α, and ELA-2 in the DHCA group were significantly higher than those in the DHCA+UTI-L group and DHCA+UTI-H group (*P *< 0.001), while the concentration of IL-10 was significantly lower in the DHCA group compared to the other 2 groups (*P *< 0.001). Compared to the DHCA+UTI-H group, the concentrations of IL-6 and TNF-α in the DHCA+UTI-L group were significantly higher (*P *< 0.01), while the concentrations of IL-10 in the DHCA+UTI-L group were significantly lower (*P *< 0.05). The LMEM results demonstrated the DHCA+UTI-L and DHCA+UTI-H groups exhibited significant differences for levels of inflammatory factors compared to the DHCA group. The low-dose UTI group significantly suppressed IL-6 levels (T4: −0.35 [−0.57, −0.13] *P *= 0.003; T5: −0.35 [−0.57, −0.13] *P *= 0.003), with the high-dose group showing more pronounced inhibition (T4: −0.78 [−1.00, −0.56] *P *< 0.001; T5: −1.24 [−1.46, −1.02] *P *< 0.001), demonstrating a dose-dependent effect. The DHCA+UTI-L group significantly increased IL-10 levels at both T4 and T5 points (T4: 1.09 [0.78, 1.40] *P *< 0.001; T5: 0.65 [0.33, 0.96] *P *< 0.001), with DHCA+UTI-H exhibiting a stronger enhancing effect (T4: 1.54 [1.23, 1.86] *P *< 0.001; T5: 1.01 [0.70, 1.32] *P *< 0.001). Similarly, UTI significantly suppressed TNF-α levels (T4: −0.47 [−0.72, −0.22] *P *< 0.001; T5: −0.63 [−0.88, −0.38] *P *< 0.001), and this inhibitory effect was more pronounced at higher doses (T4: −0.84 [−1.09, −0.59] *P *< 0.001; T5: −1.03 [−1.28, −0.78] *P *< 0.001). The DHCA+UTI-H group also exhibited some inhibitory effect on ELA-2 levels.

### Pathological findings

**Figure 5.**
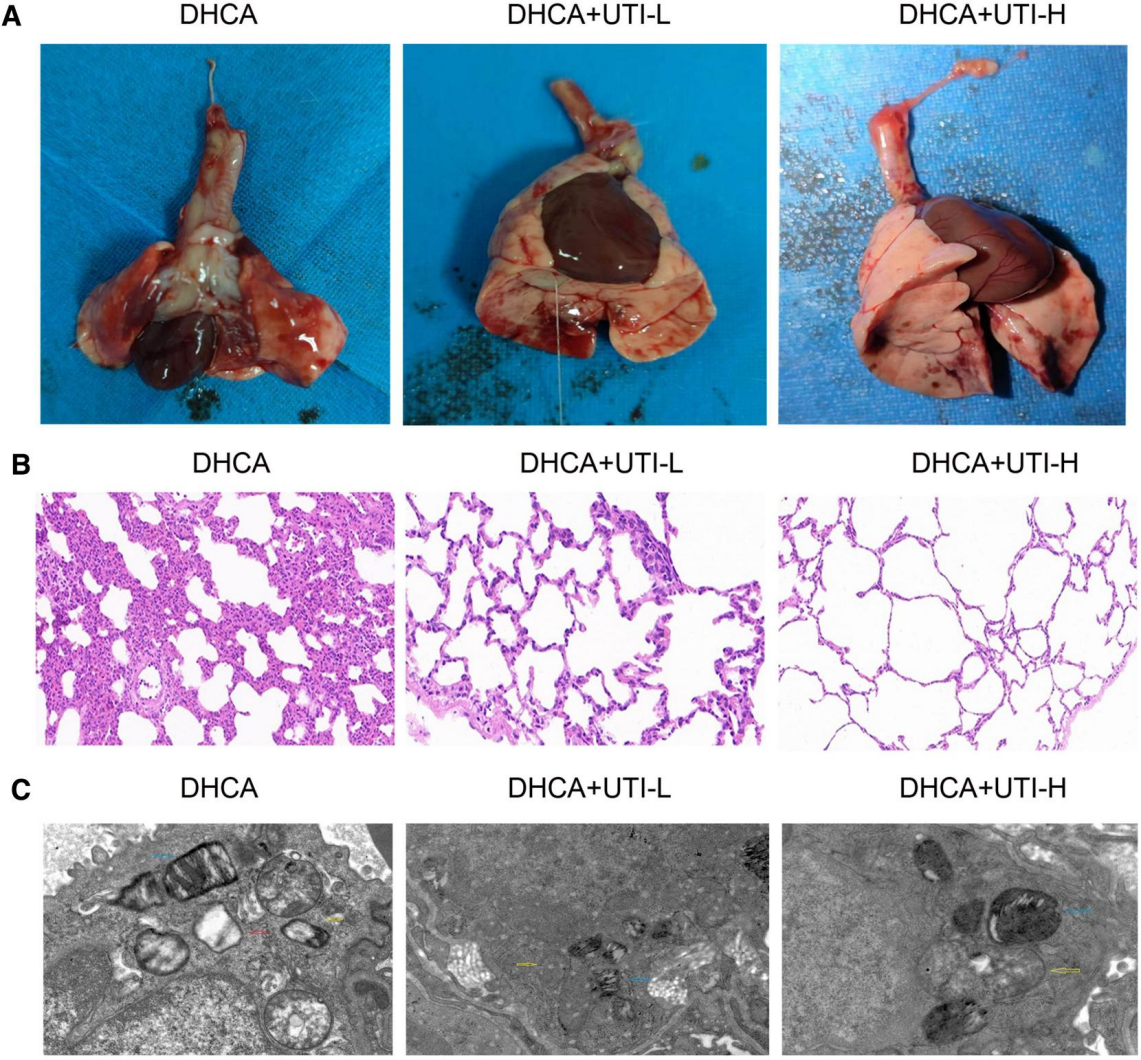
Pathological Findings of Lung Injury After UTI Treatment. (A) Analysis of lung injury in each group. (B) Representative haematoxylin and eosin staining results of lung tissues in DHCA, DHCA+UTI-L, and DHCA+UTI-H groups of rats. (C) Transmission electron microscopy results in different groups. Abbreviations: DHCA: deep hypothermic circulatory arrest; UTI: ulinastatin; UTI-L: low dose UTI treated; UTI-H: high dose UTI-treated

As shown in **Figure 5A**, compared with the DHCA group, significantly improved in lung injury were observed in UTI groups, especially in the high dose, and there was a visually reduced pulmonary oedema and inflammatory cell infiltration. Consistent findings were found from representative HE staining of lung tissues (**Figure 5B**). Compared with the DHCA group, significantly improved in lung injury were observed in UTI groups, especially in the high dose. In the DHCA group, the normal alveolar structure was destroyed, and focal necrosis and thickening of the lung interstitium were observed. Extensive infiltration of neutrophil-dominated inflammatory cells, oedema fluid in the alveolar cavity, and red blood cell and inflammatory cell exudation were also seen. Compared to the DHCA group, the pathological changes in lung histopathology were significantly reduced in the DHCA+UTI-L group, with mild-moderate pulmonary interstitial oedema and fewer inflammatory cells and erythrocyte infiltration. The alveolar wall and pulmonary interstitial capillaries were intact in the DHCA+UTI-H, with only a small amount of inflammatory cell infiltration in the pulmonary interstitium and no significant cell or fluid exudation in the alveolar cavity.

TEM results showed that mitochondria in the DHCA group exhibited high oedema, with cristae dissolution or even disappearance, reduced content of lamellar bodies, and visible formation of autophagosomes. On the contrary, the UTI group showed relatively less damage to alveolar cells, mild swelling and relatively intact mitochondria, relatively intact contents in the lamellar body, and no obvious autophagosomes were observed (**Figure 5C**).

## DISCUSSION

Acute inflammation is considered a crucial component in the pathological process following DHCA and was primarily provoked during the reperfusion phase.^19^^,^^23^ The activation of monocytes and macrophages leads to leucocytosis and the secretion of cytokines TNF-α and IL-1β, which are the initial signs of SIRS, followed by an increase in plasma IL-6 levels. TNF-α not only contributes to the release of IL-6 and IL-8 during IR injury but also reduces vascular tone and cardiac contractility,^11^ which are key components of the SIR cytokine cascade. Neutrophils are the main contributors to tissue damage in many inflammatory response diseases. The interaction between reactive oxygen species and proteases released by activated neutrophils can cause damage to endothelial cells.^24^ Elastase, a highly cytotoxic protease, can degrade connective tissue components and increase the risk of capillary leakage, leading to increased permeability of alveolar epithelial cells and pulmonary capillary endothelial cells, which can result in acute respiratory distress syndrome.^25^^,^^26^ Anti-inflammatory cytokines also play an equally important role in host defense. For IL-10, it can suppress the production of pro-inflammatory cytokines and inhibit the interaction between neutrophils and endothelial cells.

In this study, we observed that the levels of IL-6, IL-10, TNF-α, and ELA-2 at T5 in each group were significantly higher than those at T1. These data suggested that DHCA led to tissue and organ damage, resulting in an increase in inflammatory factors, which was consistent with previous study.^27^ During rewarming and reperfusion following DHCA, there was a significant increase in IL-6 and TNF-α levels in all animals compared to pre-DHCA time points. Notably, UTI administration remarkably reduced the inflammatory response, manifested by a significant decrease in IL-6, TNF-α, and an increase in IL-10. This has also been found in other studies. Nakanishi et al^28^ reported that UTI significantly improved the pulmonary function after CPB by reducing the elevation of TNF-a, IL-8, and IL-6 through administration of UTI during on-pump coronary artery revascularization. Liu et al^18^ found that UTI can reduce the release of inflammatory biomarkers such as IL-2 and TNF-α after CPB heart surgery. From the above findings, it can be inferred that UTI treatment can suppress proinflammatory cytokine elevation and upregulate the release of anti-inflammatory mediators in CPB with DHCA.

Strong evidence demonstrates the considerable anti-inflammatory effect of UTI. However, the dosage of UTI varies widely in animal experiments and human clinical studies, and the timing and dosage may affect its efficacy. In patients undergoing cardiac surgery, UTI doses range from 5000 to 6 × 10^4^ U/kg. In Nakanishi et al’s^28^ study, 5000 U/kg of UTI was used, and his team found that prepump administration of urinary trypsin inhibitor attenuates the elevation of IL-6 and IL-8 release immediately after CPB. In Xu et al’s^17^ research, they reported that 20 000 U/kg of UTI attenuated the elevation of cytokines and PMNE, reduced the pulmonary injury, and improved the pulmonary function after CPB under DHCA. Findings from Liu et al’s^18^ team revealed that the higher dose (6 × 10^4^ U/kg) of UTI resulted in higher blood concentration and a better therapeutic effect compared with lower dose of UTI (2, 4 × 10^4^ U/kg) in a dose-dependent manner. To investigate whether higher doses of UTI can bring more benefits, we set up 10 × 10^4^ U/kg UTI group. Fortunately, 4 hours after CPB under DHCA, 10 × 10^4^ U/kg of UTI significantly reduced levels IL-6 and TNF-α and sharply increased IL-10 level as comparison to the 5 × 10^4^ U/kg group. This conveys a signal that higher UTI levels may bring more benefits. Our rat animal model provided standardized injury severity and separately evaluated the response of UTI as a single factor to inflammatory factors and lung tissue injury, which is also a major advantage of the study.

The decrease in TNF-α and increase in IL-10 levels were beneficial for inhibiting the inflammatory reaction and maintaining haemodynamic stability.^28^ Our haemodynamic findings demonstrated that the DHCA group exhibited significantly impaired cardiovascular recovery compared to UTI-treated animals, with persistently lower heart rate and blood pressure. This may be due to complete cardiac ischaemia during DHCA, leading to mitochondrial dysfunction, a burst of reactive oxygen species, and vascular endothelial damage.^29^ Additionally, the haematocrit levels also showed significant abnormalities in the DHCA group, with significantly lower levels compared to the UTI groups at T3, T4, and T5. This may be attributed to inflammation-induced endothelial damage, increased capillary permeability, and fluid shift to the interstitial space. To maintain circulation, a large amount of fluid was administered during the experiment. The most notable changes observed in the metabolites affected the glucose and lactate levels. Here, UTI was found to be significantly lower lactate concentrations and higher blood glucose levels compared to the DHCA group at T3, T4, and T5. Meanwhile, we found that the oxygenation index of the drug group was significantly improved than the DHCA group after rewarming, suggesting that UTI may improve tissue perfusion and reduce hypoxia. Further, we noticed that the pathological changes in lung tissue were significantly reduced in the UTI groups, especially in the high concentration UTI group, where the alveolar walls and interstitial capillaries remained intact. This may be attributed to UTI reducing inflammatory response, correcting ventilation/perfusion ratio dysfunction, improving pulmonary oxygenation function, and further alleviating lung injury caused by CPB under DHCA. We recognize that the pulmonary trauma in our rats DHCA model appeared more severe than typically observed clinically. This might be attributed to the lack of clinical auxiliary measures, such as anterograde cerebral perfusion.^30^ However, the histological patterns closely mirror clinical findings in fatal ARI cases after cardiac surgery.^5^ This study provides a pathological model for exploring DHCA-related lung injury.

Several limitations were present in this study. First, the limited blood volume in rats and financial constraints restricted our ability to detect proteins associated with cellular signalling and inflammatory pathways, resulting in a lack of detailed understanding of the molecular mechanisms of UTI. Second, to reduce blood loss, cardiac arrest was achieved through deep hypothermia without the use of cardioplegic solution. Third, since none of the rats in this study had experienced thoracotomy and were healthy, the inflammatory stimulation observed may differ from that in clinical settings. Additionally, the highest tested UTI dose was 10 × 10^4^ U/kg; further studies are needed to assess whether higher doses could enhance anti-inflammatory effects or further mitigate lung injury. Future studies will integrate pulmonary function parameters both in advanced animal models and clinical biomarker studies to establish links between UTI and clinically meaningful pulmonary protection. It will also be considered to combine different ventilation strategies with UTI to explore the impact on postoperative respiratory dysfunction.

## CONCLUSIONS

5 × 10^4^ U/kg and 10 × 10^4^ U/kg of UTI could safely and effectively reduce the inflammatory response and lung tissue injury after CPB under DHCA. For conditions where therapeutic effects are not achieved at low dose, 10 × 10^4^ U/kg may be a promising therapeutic dose.

## Supplementary Material

ivaf177_Supplementary_Data

## Data Availability

The data supporting this study’s findings are available from the corresponding author upon reasonable request.
