# Supplementary material for: Effects of Ulinastatin on Inflammation Response and Lung Tissue Injury in Deep Hypothermic Circulatory Arrest
Source: Interdiscip Cardiovasc Thorac Surg. 2025 Jul 29;40(9):ivaf177. doi: 10.1093/icvts/ivaf177 (PMC12401673; doi:10.1093/icvts/ivaf177)
Supplement: ivaf177_Supplementary_Data [file ivaf177_supplementary_data.pdf]

Supplementary Table 1 Vasoactive drugs and fluid dose management

		1	2	3	4	5	6
Norepinephrine (μg/kg)	DHCA	2	5	2	3	2	6
	DHCA+UTI-L	3	3	4	2	3	1
	DHCA+UTI-H	1	2	3	4	5	2
Gelofusine (mL)	DHCA	16	15	20	9	15	13
	DHCA+UTI-L	8	12	5	11	10	9
	DHCA+UTI-H	9	10	6	8	11	12

DHCA, deep hypothermic circulatory arrest; UTI, ulinastatin; UTI-L, low dose UTI-treated; UTI-H, high dose UTI-treated.

Supplementary Table 2 The quantitative values of inflammatory factors at different time points among the three groups

**Group**		**IL-6 (pg/mL)**	**IL-10 (pg/mL)**	**TNF-α (pg/mL)**	**ELA2 (ng/mL)**
	**T2**	71.79 (64.96, 81.34)	32.52 (29.90, 35.90)	34.00 (32.25, 40.42)	0.50 (0.47, 0.53)
**DHCA**	**T4**	3055.99 (2432.82, 3191.13)	160.00 (127.99, 176.59)	227.11 (197.06, 233.47)	1.96 (1.85, 2.14)
	**T5**	8921.95 (86.14.11, 9507.79)	979.94 (901.44, 1059.72)	623.5 (581.03, 665.29)	5.13 (4.60, 5.34)
**DHCA+UTI-L**	**T2**	62.06 (59.82, 70.30)	33.63 (30.15, 37.21)	35.51 (28.58, 42.32)	0.27 (0.21, 0.33)
	**T4**	1792.71 (1552.94, 2027.98)	594.54 (488.11, 657.90)	136.60 (128.74, 141.32)	1.57 (1.45, 1.83)
	**T5**	6010.11 (5224.69, 6650.57)	1960.22 (1852.03, 2110.03)	312.94 (308.19, 336.42)	3.57 (3.40, 3.94)
**DHCA+UTI-H**	**T2**	67.60 (62.18, 72.71)	37.30 (34.65, 39.38)	36.27 (32.45, 39.28)	0.44 (0.28, 0.52)
	**T4**	1173.63 (1094.18, 1368.79)	883.71 (793.33, 920.13)	94.20 (81.09, 103.95)	1.13 (1.09, 1.19)
	**T5**	2310.24 (2077.86, 2735.68)	3041.67 (2837.53, 3158.02)	219.28 (201.29, 248.42)	3.19 (3.13, 3.35)

The data in the table represent the median (interquartile range [IQR]).

DHCA, deep hypothermic circulatory arrest; UTI, ulinastatin; UTI-L, low dose UTI-treated; UTI-H, high dose UTI-treated; IL-6, interleukin-6; IL-10, interleukin-10; TNF-α, tumor necrosis factor-α; ELA, neutrophil elastase.

Supplementary Table 3 The quantitative values of relevant indicators at different time points among the three groups

Group		Glucose (mg/dL)	Lactate (mmol/L)	Hematocrit (%)	Oxygenation index (%)
DHCA	**T1**	200.50 (187.75, 222.25)	0.65 (0.48, 0.75)	37.00 (35.75, 38.25)	513.00 (430.00, 543.50)
	**T2**	211.00 (202.50, 228.00)	0.61 (0.43, 0.88)	20.50 (19.00, 22.00)	406.50 (373.50, 437.00)
	**T3**	440.50 (363.25, 497.00)	5.38 (4.93, 5.80)	15.00 (13.75, 16.00)	306.00 (284.00, 320.00)
	**T4**	519.50 (486.25, 562.00)	5.53 (5.20, 7.21)	16.50 (16.00, 17.50)	240.00 (225.00, 262.75)
	**T5**	517.50 (444.00, 535.00)	4.98 (4.73, 6.21)	18.50 (18.00, 19.25)	259.50 (231.25, 281.50)
DHCA+UTI-L	**T1**	214.50 (207.75, 233.75)	0.55 (0.49, 0.63)	34.00 (31.75, 37.50)	472.50 (451.00, 494.50)
	**T2**	219.00 (213.00, 222.00)	0.93 (0.68, 0.99)	20.00 (18.75, 20.00)	434.50 (423.50, 463.00)
	**T3**	316.00 (315.25, 340.00)	3.45 (2.89, 3.81)	19.00 (18.00, 20.00)	395.00 (351.00, 404.50)
	**T4**	410.00 (399.00, 424.25)	2.95 (1.35, 3.35)	22.50 (19.75, 23.00)	381.50 (313.00, 427.25)
	**T5**	354.50 (275.50, 371.25)	3.08 (2.14, 3.17)	24.00 (22.75, 24.25)	374.50 (307.75, 459.50)
DHCA+UTI-H	**T1**	197.50 (194.50, 211.75)	0.76 (0.68, 0.93)	36.50 (32.75, 39.00)	487.00 (455.25, 523.00)
	**T2**	205.00 (177.25, 238.75)	0.90 (0.69, 1.00)	17.50 (15.75, 21.25)	451.50 (393.75, 480.25)
	**T3**	325.00 (288.25, 370.25)	3.65 (3.39, 4.08)	18.50 (17.00, 21.25)	419.00 (388.50, 445.75)
	**T4**	389.00 (339.50, 402.25)	2.53 (2.38, 2.99)	21.00 (19.75, 22.00)	418.00 (367.50, 454.50)
	**T5**	302.50 (257.75, 324.00)	2.53 (2.29, 2.79)	23.50 (20.75, 25.00)	437.50 (397.00, 471.50)

The data in the table represent the median (interquartile range [IQR]).

DHCA, deep hypothermic circulatory arrest; UTI, ulinastatin; UTI-L, low dose UTI-treated; UTI-H, high dose UTI-treated.

Supplementary Table 4 The quantitative values of relevant indicators at different time points among the three groups

Group		Heart rate (bpm)	Blood pressure (mmHg)
	**T1**	321.00 (317.75, 325.75)	94.50 (92.75, 96.25)
	**T2**	313.50 (310.50, 316.75)	63.50 (61.50, 68.00)

**DHCA**	**T3**	191.50 (187.25, 197.00)	45.50 (41.75, 47.00)
	**T4**	270.50 (245.50, 279.00)	73.50 (71.00, 76.00)
	**T5**	265.00 (246.75, 277.00)	70.50 (66.25, 72.25)
	**T1**	321.00 (318.75, 325.50)	94.50 (92.00, 98.25)
	**T2**	321.00 (319.75, 325.25)	69.00 (67.00, 71.00)
**DHCA+UTI-L**	**T3**	240.50 (232.75, 249.50)	49.50 (47.75, 56.50)
	**T4**	321.50 (319.50, 324.25)	82.00 (79.50, 83.75)
	**T5**	315.50 (310.75, 323.00)	94.00 (91.50, 96.00)
	**T1**	322.50 (317.75, 330.25)	97.00 (95.75, 99.50)
	**T2**	316.50 (314.50, 319.25)	70.00 (69.00, 71.50)
**DHCA+UTI-H**	**T3**	240.50 (233.75, 256.50)	54.50 (52.00, 60.25)
	**T4**	322.00 (319.00, 329.25)	83.50 (82.00, 85.00)
	**T5**	321.50 (316.50, 323.50)	95.00 (94.25, 96.25)

The data in the table represent the median (interquartile range [IQR]).

DHCA, deep hypothermic circulatory arrest; UTI, ulinastatin; UTI-L, low dose UTI-treated; UTI-H, high dose UTI-treated.

Supplementary Table 5 The analysis results of the main effects and group-time interaction effects of different variables based on the linear mixed-effects model

**Variables**	Oxygenation index (%)	Glucose (mg/dL)	Lactate (mmol/L)	Hematocrit (%)	Heart rate (bpm)	Blood pressure (mmHg)
**Predictors**	Estimate (95%CI)	Estimate (95%CI)	Estimate (95%CI)	Estimate (95%CI)	Estimate (95%CI)	Estimate (95%CI)
(Intercept)	6.19*** (5.97, 6.40)	5.34*** (5.19, 5.48)	-0.49 (-0.73, -0.24)	3.59*** (3.50, 3.68)	5.77*** (5.70, 5.85)	4.55 (4.49, 4.61)
T2	-0.18* (-0.33, -0.03)	-1.66×10^{-3} (-0.19, 0.18)	-0.06 (-0.37, 0.25)	-0.59*** (-0.68, -0.49)	-0.02 (-0.06, 0.02)	-0.38*** (-0.44, -0.31)
T3	-0.50***	0.71***	2.14***	-0.90***	-0.51***	-0.78***

	(-0.65, -0.34)	(0.52, 0.89)	(1.83, 2.45)	(-1.00, -0.81)	(-0.56, -0.47)	(-0.84, -0.71)
T4	-0.76***	0.91***	2.34***	-0.77***	-0.20***	-0.25***
	(-0.91, -0.61)	(0.73, 1.10)	(2.03, 2.65)	(-0.86, -0.67)	(-0.24, -0.16)	(-0.31, -0.18)
T5	-0.61***	0.79***	2.19***	-0.67***	-0.20***	-0.31***
	(-0.76, -0.46)	(0.61, 0.98)	(1.88, 2.50)	(-0.76, -0.57)	(-0.24, -0.16)	(-0.37, -0.24)
UTI-L	-3.27×10^{-3}	0.04	-0.05	-0.05	-2.40×10^{-3}	4.85×10^{-3}
	(-0.30, 0.30)	(-0.16, 0.24)	(-0.40, 0.30)	(-0.18, 0.07)	(-0.10, 0.11)	(-0.08, 0.09)
UTI-H	0.02	-0.02	0.21	-0.01	0.01	0.03
	(-0.28, 0.32)	(-0.23, 0.18)	(-0.14, 0.56)	(-0.14, 0.12)	(-0.10, 0.11)	(-0.06, 0.12)
T2:UTI-L	0.06	-0.02	0.41	-2.40×10^{-3}	0.02	0.05
	(-0.16, 0.27)	(-0.28, 0.24)	(-0.03, 0.85)	(-0.14, 0.13)	(-0.04, 0.08)	(-0.04, 0.15)
T3:UTI-L	0.23*	-0.29*	-0.37	0.31***	0.22***	0.16***
	(0.01, 0.44)	(-0.55, -0.03)	(-0.81, 0.07)	(0.18, 0.44)	(0.16, 0.28)	(0.07, 0.26)
T4:UTI-L	0.47***	-0.28*	-0.96***	0.29***	0.20***	0.10*
	(0.26, 0.69)	(-0.54, -0.02)	(-1.40, -0.52)	(0.16, 0.43)	(0.14, 0.26)	(0.01, 0.19)
T5:UTI-L	0.33**	-0.41**	-0.65**	0.29***	0.18***	0.30***
	(0.12, 0.55)	(-0.67, -0.15)	(-1.09, -0.21)	(0.16, 0.43)	(0.12, 0.24)	(0.20, 0.39)
T2:UTI-H	0.06	-0.03	0.10	-0.08	3.34×10^{-3}	0.04
	(-0.15, 0.28)	(-0.29, 0.24)	(-0.34, 0.54)	(-0.21, 0.06)	(-0.05, 0.06)	(-0.05, 0.13)
T3:UTI-H	0.31**	-0.25	-0.62**	0.28***	0.24***	0.20***
	(0.09, 0.52)	(-0.51, 0.01)	(-1.06, -0.18)	(0.15, 0.42)	(0.18, 0.29)	(0.11, 0.29)
T4:UTI-H	0.56***	-0.28*	-1.10***	0.24***	0.20***	0.08
	(0.34, 0.77)	(-0.54, -0.02)	(-1.54, -0.66)	(0.11, 0.38)	(0.14, 0.26)	(-0.01, 0.17)
T5:UTI-H	0.48***	-0.48***	-0.98***	0.25***	0.19***	0.28***
	(0.26, 0.69)	(-0.74, -0.21)	(-1.42, -0.54)	(0.12, 0.38)	(0.13, 0.25)	(0.19, 0.37)

$^{*}P<0.05$, $^{**}P<0.01$, $^{***}P<0.001$

Supplementary Table 6 The analysis results of the main effects and group-time interaction effects of inflammatory factors based on the linear mixed-effects model

**Variables**	IL-6 (pg/mL)	IL10 (pg/mL)	TNF-α (pg/mL)	ELA-2 (ng/mL)
**Predictors**	Estimate (95%CI)	Estimate (95%CI)	Estimate (95%CI)	Estimate (95%CI)
(Intercept)	4.27*** (4.15, 4.39)	3.47*** (3.27, 3.66)	3.55*** (3.39, 3.70)	-0.69*** (-0.89, -0.49）
T4	3.66*** (3.50, 3.81)	1.59*** (1.37, 1.81)	1.86*** (1.68, 2.04)	1.33*** (1.06, 1.60)
T5	4.83*** (4.67, 4.98)	3.41*** (3.19, 3.63)	2.90*** (2.72, 3.07)	2.28*** (2.01, 2.55)
UTI-L	-0.09 (-0.26, 0.08)	0.08 (-0.19, 0.36)	-0.03 (-0.25, 0.19)	-0.63*** (-0.91, -0.35)
UTI-H	-0.05 (-0.22, 0.12)	0.13 (-0.14, 0.41)	-0.02 (-0.24, 0.19)	-0.24 (-0.52, 0.04)
T4:UTI-L	-0.35** (-0.57, -0.13)	1.09*** (0.78, 1.40)	-0.47*** (-0.72, -0.22)	0.49* (0.11, 0.86)
T5:UTI-L	-0.35** (-0.57, -0.13)	0.65*** (0.33, 0.96)	-0.63*** (-0.88, -0.38)	0.33 (-0.05, 0.71)
T4:UTI-H	-0.78*** (-1.00, -0.56)	1.54*** (1.23, 1.86)	-0.84*** (-1.09, -0.59)	-0.28 (-0.66, 0.10)
T5:UTI-H	-1.24*** (-1.46, -1.02)	1.01*** (0.70, 1.32)	-1.03*** (-1.28, -0.78)	-0.17 (-0.55, 0.21)

*P<0.05, **P<0.01, ***P<0.001
